# Estimating the Incidence of Influenza at the State Level — Utah, 2016–17 and 2017–18 Influenza Seasons

**DOI:** 10.15585/mmwr.mm6850a2

**Published:** 2019-12-20

**Authors:** Michelle M. Hughes, Anna E. Carmack, Keegan McCaffrey, Melanie Spencer, Gregg M. Reed, Mary Hill, Angela Dunn, Ilene Risk, Shikha Garg, Carrie Reed, Matthew Biggerstaff, Jeanmarie Mayer, Per Gesteland, Kent Korgenski, Kristin Dascomb, Andrew Pavia, Melissa A. Rolfes

**Affiliations:** ^1^Epidemic Intelligence Service, CDC; ^2^National Center for Immunization and Respiratory Diseases, CDC; ^3^University of Maryland Medical Center, Baltimore, Maryland; ^4^Utah Department of Health; ^5^Salt Lake County Health Department, Salt Lake City, Utah; ^6^University of Utah Health, Salt Lake City, Utah; ^7^Intermountain Healthcare, Salt Lake City, Utah.

The 2017–18 U.S. influenza season was notable for its high severity, with approximately 45 million illnesses and 810,000 influenza-associated hospitalizations throughout the United States (*1*). The purpose of the investigation reported here was to create a state-level estimate of the number of persons in Utah who became ill with influenza disease during this severe national seasonal influenza epidemic and to create a sustainable system for making timely updates in future influenza seasons. Knowing the extent of influenza-associated illness can help public health officials, policymakers, and clinicians tailor influenza messaging, planning, and responses for seasonal influenza epidemics or during pandemics. Using national methods and existing influenza surveillance and testing data, the influenza burden (number of influenza illnesses, medical visits for influenza, and influenza-associated hospitalizations) in Utah during the 2016–17 and 2017–18 influenza seasons was estimated. During the 2016–17 season, an estimated 265,000 symptomatic illnesses affecting 9% of Utah residents occurred, resulting in 125,000 medically attended illnesses and 2,700 hospitalizations. During the 2017–18 season, an estimated 338,000 symptomatic illnesses affecting 11% of Utah residents occurred, resulting in 160,000 medically attended illnesses and 3,900 hospitalizations. Other state or county health departments could adapt similar methods in their jurisdictions to estimate the burden of influenza locally and support prompt public health activities.

Since the 2009 influenza pandemic, CDC has estimated the burden of influenza in the United States each year (*1*,*2*). However, influenza activity can vary widely across the country, making the use of national burden estimates difficult for state or county public health messaging, planning, and responses (*3*). The 2017–18 influenza season was one of high severity, with an estimated 45 million illnesses, 21 million medically attended illnesses, 810,000 hospitalizations, and 61,000 deaths occurring throughout the United States (*1*). At the peak of influenza activity in early 2018, CDC partnered with the Utah Department of Health and the Salt Lake County Health Department to conduct a rapid assessment of the burden of influenza in Utah and to determine the applicability of national methods at a state and county level.

In February 2018, a field investigation was conducted in Utah to rapidly gather local data to estimate the burden of influenza during the 2016–17 influenza season and midway through the 2017–18 season. Updated data from the entire 2017–18 season (rather than midseason data used for real-time estimates) were incorporated into the estimates presented here. To estimate burden, national multipliers were used to extrapolate the rate of influenza-associated hospitalizations to the rates of symptomatic illnesses in the community and medically attended illnesses, as has been previously described (*4*). The analysis was restricted to hospitalizations reported during the 2016–17 and 2017–18 influenza seasons (surveillance weeks 40–20). Hospitalization rates were calculated by five age groups (0–4, 5–17, 18–49, 50–64, and ≥65 years) using contemporary population data obtained from the National Center for Health Statistics bridged-race population estimates. In Utah, hospitalizations with laboratory-confirmed influenza are reportable (*5*); however, surveillance for influenza-associated hospitalizations in Utah depends on clinician-ordered testing and thus underestimates the actual rate of influenza hospitalizations. To adjust reported hospitalization rates for underdetection of influenza, as is done nationally, CDC, the Utah Department of Health, and the Salt Lake County Health Department coordinated with two large health care systems in Utah to extract data on whether a patient hospitalized with a diagnosis of acute respiratory illness (based on *International Classification of Diseases, Tenth Edition* discharge codes) (Supplementary Table 1, https://stacks.cdc.gov/view/cdc/83482) was tested for influenza and what type of influenza test was performed. Data were extracted for acute respiratory hospitalizations occurring during December 2016–April 2017 and December 2017–April 2018. Pooled sensitivity was calculated for influenza tests performed, after assigning sensitivities from a literature review (*6*) and using the most sensitive test result if multiple tests were performed. Adjustments for underdetection were calculated as the inverse of the probability of being tested multiplied by the pooled test sensitivity and were applied to crude hospitalization rates.

To estimate the number of influenza-associated hospitalizations in Utah, the adjusted hospitalization rates were applied to age group–specific population estimates. Using national multipliers, the number of symptomatic illnesses was estimated using previously published age group–specific ratios of the number of illnesses that one hospitalization represents (*4*). Age-stratified data on national care-seeking behavior from the Behavioral Risk Factor Surveillance System were used to estimate the number of persons with symptomatic influenza-like illnesses who sought medical care (Supplementary Table 2, https://stacks.cdc.gov/view/cdc/83483) (*4*). Age group–specific estimates were summed to obtain the influenza burden in Utah for each season. The 95% confidence intervals around the burden estimates were constructed using a combination of standard error of the hospitalization rate (Poisson distribution), percentage tested (binomial), and pooled test sensitivity (binomial), assuming independence of parameters. Symptomatic illnesses and medically attended illnesses were estimated to three significant figures, and hospitalizations to two significant figures. Analyses were conducted using SAS statistical software (version 9.4; SAS Institute). An analytic tool was provided by CDC to the state and county health departments to facilitate future estimations.

The overall crude rate of influenza-associated hospitalizations in Utah was 47 per 100,000 persons during the 2016–17 influenza season and 71 per 100,000 during the 2017–18 season. Based on influenza testing data, 58% of persons hospitalized with acute respiratory illnesses were tested for influenza during the 2016–17 season, and 63% were tested during the 2017–18 season; this increase was primarily driven by increased testing among children (Table). Across seasons, tests for influenza virus RNA were the most common tests used (91%), followed by rapid antigen tests (8%).

**TABLE:** Estimated numbers of influenza-associated illnesses, medically attended illnesses, and hospitalizations — Utah, 2016–17 and 2017–18 influenza seasons

Age group (yrs)	Population*	No. of influenza-associated hospitalizations	Unadjusted hospitalization rate^†^	% Tested	Average % sensitivity	Underdetection multiplier	Adjusted hospitalization rate^†^	Estimated burden no. (95% CI)^§^		
								Illnesses	Medically attended illnesses	Hospitalizations
**2016–17 influenza season**										
0–4	253,338	96	38	42	92	2.56	97	35,300 (25,600–45,000)	23,700 (17,200–30,100)	250 (180–310)
5–17	666,090	59	9	31	92	3.48	31	74,900 (47,400–102,000)	39,000 (24,700–53,300)	210 (130–280)
18–49	1,362,564	233	17	43	94	2.47	42	103,000 (86,900–118,000)	38,000 (32,200–43,800)	580 (490–660)
50–64	441,528	247	56	65	94	1.62	91	37,800 (32,400–43,200)	16,200 (13,900–18,600)	400 (340–460)
≥65	320,801	784	244	76	80	1.63	399	14,100 (12,900–15,300)	7,880 (7,220–8,540)	1,300 (1,200–1,400)
**Total**	**3,044,321**	**1,419**	**47**	**58**	**—**	**—**	**89**	**265,000 (205,000–324,000)**	**125,000 (95,100–154,000)**	**2,700 (2,300–3,100)**
**2017–18 influenza season**										
0–4	255,200	168	66	55	93	1.95	128	46,900 (38,100–55,800)	31,500 (25,500–37,400)	330 (270–390)
5–17	671,499	129	19	57	93	1.89	36	89,100 (70,000–108,000)	46,300 (36,400–56,300)	240 (190–300)
18–49	1,393,111	292	21	47	93	2.30	48	120,000 (104,000–136,000)	44,300 (38,300–50,300)	670 (580–760)
50–64	446,451	388	87	66	92	1.64	142	60,000 (52,800–67,100)	25,800 (22,700–28,900)	640 (560–710)
≥65	335,572	1,214	362	78	78	1.65	598	22,100 (20,500–23,600)	12,400 (11,500–13,200)	2,000 (1,900–2,100)
**Total**	**3,101,833**	**2,191**	**71**	**63**	**—**	**—**	**125**	**338,000 (285,000–391,000)**	**160,000 (134,000–186,000)**	**3,900 (3,500–4,300)**

**Abbreviation:** CI = confidence interval.

* Population estimates from Utah's Public Health Indicator Based Information System are vintage 2017 U.S. Census Bureau July 1 estimates based on the 2010 census counts (http://ibis.health.utah.gov/query/result/pop/PopMain/Count.htm). The estimates were produced by the U.S. Census Bureau’s Population Estimates Program in collaboration with the National Center for Health Statistics and released in June 2018.

^†^ Hospitalizations per 100,000 population.

^§^ CIs were calculated from the combined standard error of the hospitalization rate, percentage tested, and pooled test sensitivity, assuming independence. If these parameters are not independent, the assumption will result in an underestimation of the variability and tighter CIs.

Based on the adjusted influenza hospitalization rates in Utah and using national multipliers, an estimated 265,000 symptomatic illnesses (affecting 9% of Utah residents), 125,000 medically attended illnesses, and 2,700 hospitalizations occurred in Utah during the 2016–17 influenza season (Table). During 2017–18, an estimated 338,000 symptomatic illnesses (among 11% of Utah residents), 160,000 medically attended illnesses, and 3,900 hospitalizations occurred in the state. In both seasons, the majority of symptomatic and medically attended illnesses occurred in persons aged 5–17 and 18–49 years. Adults aged ≥65 years accounted for 47% of estimated hospitalizations during 2016–17 and 52% during the 2017–18 season.

## Discussion

National methods were adopted to quantify the number of persons in Utah who were ill, sought medical care, or were hospitalized with influenza during the 2016–17 and 2017–18 influenza seasons. Influenza affected an estimated 9% of Utah residents during the 2016–17 season and an estimated 11% during the 2017–18 season; hundreds of thousands of medical visits and thousands of hospitalizations occurred, with higher numbers during the high-severity 2017–18 season (*7*), compared with the 2016–17 season. The Utah Department of Health and the Salt Lake County Health Department used these methods to synthesize influenza surveillance data, and the Salt Lake County Health Department published weekly estimates of state-level influenza burden during the 2018–19 and current influenza seasons (*8*). These timely subnational estimates at the state and county levels are valuable for providing a local understanding of influenza activity given the geographic variation in influenza activity in the United States (*3*).

Beyond those described in this analysis, other methods have also been used to estimate state-specific burden of influenza, including those that use hospital discharge databases, hospital admission logs, and community surveys (*9*,*10*). Various approaches are used to estimate state-level disease incidence, and states can adapt those methods most suitable for the data sets routinely available in their jurisdiction. In Utah, for example, hospitalizations are reportable, and the existing close relationship between public health departments and health systems allows rapid access to information for adjusting hospitalization rates for local influenza testing patterns.

The findings in this report are subject to at least three limitations, all related to the previously described national burden estimation methods (*2*,*4*). First, the hospitalization underdetection multiplier does not account for persons with cases of influenza without an *International Classification of Diseases* code for acute respiratory illness or for recent improvements in influenza-specific assays, which might have increased influenza test sensitivities (*2*). Second, the adjusted hospitalization rates are likely underestimated because the methods did not account for the underreporting of hospitalized influenza illnesses to health departments in Utah. Finally, the multipliers used to extrapolate rates of hospitalization to illnesses and of illnesses to medically attended illness were based on previous seasons’ data and were not specific to Utah (*4*).

Nationally, annual estimates of influenza disease burden have been useful for communicating the importance of influenza as a public health concern, describing the variability of influenza from season to season, and assessing the impact of public health interventions such as vaccination (*4*). Other state and county public health officials, policymakers, and health care practitioners might find more geographically targeted burden estimates useful for improved communications, public health response, and resource planning and allocation during seasonal epidemics and pandemics. CDC continues to develop resources to support local assessments of influenza burden by season; interested jurisdictions can contact CDC’s Influenza Division (404-639-3727) for more information.

**Summary**

**What is already known about this topic?**

Influenza activity can vary widely based on geographic location, and national data on the numbers of persons affected by influenza do not reflect this potential variation.

**What is added by this report?**

Application of national methods to estimate the burden of influenza at the state level found that influenza affected 9% and 11% of Utah residents during the 2016–17 and 2017–18 influenza seasons, respectively.

**What are the implications for public health practice?**

Local estimation of influenza disease burden can help public health officials, policymakers, and clinicians tailor influenza messaging, planning, and responses for their jurisdictions. State and county health departments might consider adapting these methods to their jurisdictions in future influenza seasons.
